# Fatal Triple-M Overlap Syndrome After Atezolizumab–Bevacizumab Therapy in Advanced Hepatocellular Carcinoma: A Case Report of Diagnostic Complexity and Therapeutic Escalation With Ruxolitinib

**DOI:** 10.7759/cureus.105174

**Published:** 2026-03-13

**Authors:** Jonathan Moyambi, Serge E Bambule, Alfakihi Ahmed Said IsmaIl, Marc Nzanzu Mowavingi, Adolphe M Kasongo

**Affiliations:** 1 Cardiology, Ibn Rochd University Hospital Center, Casablanca, MAR; 2 Medical Oncology, Ibn Rochd University Hospital Center, Casablanca, MAR; 3 Internal Medicine, University of Kinshasa, Kinshasa, COD; 4 Hepatology, Henri Mondor University Hospitals, Créteil, FRA; 5 Cardiology, South Francilien Hospital Center, Corbeil-Essonnes, FRA

**Keywords:** advanced hepatocellular carcinoma, case report, immune checkpoint inhibitors (icis), myasthenia-like syndrome, myocarditis, myositis, ruxolitinib, triple-m overlap syndrome

## Abstract

Immune checkpoint inhibitors (ICIs) have improved outcomes in advanced hepatocellular carcinoma (HCC) but may cause severe immune-related adverse events (irAEs). We report the case of a 78-year-old man with known ischemic heart disease and with advanced HCC, who received atezolizumab plus bevacizumab and, after the third cycle, developed dysphagia, proximal muscle weakness, and concomitant elevations in high-sensitivity cardiac troponin T (hs-cTnT) and creatine kinase (CK). Coronary angiography was unchanged compared with prior findings, and cardiac magnetic resonance imaging was negative for myocarditis. Integrating the clinical presentation with serial laboratory findings led to a diagnosis of a myocarditis-myositis-myasthenia-like (Triple-M) overlap syndrome. Initial treatment with high-dose intravenous corticosteroids, intravenous immunoglobulin, and noninvasive ventilation (NIV) for hypercapnic respiratory failure resulted in early clinical and biochemical improvement. Secondary deterioration prompted therapeutic escalation with re-initiation of intravenous corticosteroids and addition of ruxolitinib. Despite biomarker response, the course was complicated by pneumococcal septic shock, leading to fatal multiorgan failure. This case highlights the diagnostic complexity of ICI-related cardiac and neuromuscular toxicities and the importance of early multidisciplinary management. Janus kinase (JAK) inhibition may represent a rescue option in corticosteroid-refractory disease, but with an increased risk of infection.

## Introduction

Immune checkpoint inhibitors (ICIs) have reshaped outcomes across multiple solid tumors, including advanced hepatocellular carcinoma (HCC), for which atezolizumab plus bevacizumab is currently a first-line standard of care [1]. By enhancing antitumor immunity through modulation of the Programmed Cell Death Protein-1 (PD-1)/Programmed Cell Death Ligand-1 (PD-L1) axis, these agents can also trigger immune-related adverse events (irAEs) involving several organ systems. The most commonly affected sites are the gastrointestinal tract and endocrine glands, while central nervous system, cardiovascular, and musculoskeletal toxicities are less frequent but potentially life-threatening [2]. Among cardiovascular irAEs, ICI-associated myocarditis is uncommon (reported incidence <1%) but carries a high mortality rate, estimated at 40%-50% in early series [3,4]. Importantly, ICI myocarditis frequently overlaps with inflammatory myositis and, less commonly, myasthenia gravis-like features, known as Triple-M (myocarditis-myositis-myasthenia gravis) overlap syndrome. It is characterized by a rapid onset and carries markedly worse outcomes, with in-hospital mortality due to synergistic cardiac, respiratory, and neuromuscular failure [5,6].

We report the case of an elderly patient with documented ischemic heart disease treated with atezolizumab-bevacizumab for advanced HCC, who presented with inaugural dysphagia accompanied by concomitant elevations in high-sensitivity cardiac troponin T (hs-cTnT) and creatine kinase (CK). 

## Case presentation

Patient information

A 78-year-old man with no prior tobacco use and a history of chronic regular alcohol intake, presently in alcohol withdrawal. His notable medical history includes non-insulin-dependent type 2 diabetes, ischemic coronary heart disease requiring angioplasty in 2008 and 2021, and dyslipidemia; all well monitored and treated according to current guidelines. He had advanced HCC on a non-cirrhotic liver, histologically diagnosed in April 2025. The patient received first-line systemic therapy consisting of atezolizumab plus bevacizumab on a 21-day cycle schedule; the third cycle was administered in July 2025.

Clinical findings

At emergency admission following the third cycle of therapy, the patient presented with a preserved general condition and overall stable vital signs. Cardiovascular examination was unremarkable. Respiratory examination disclosed shallow tachypnea with adequate oxygen saturation on room air. Abdominal examination showed no clinical signs of liver failure or portal hypertension. Neurological examination revealed a Glasgow Coma Scale score of 15/15 [7], dysarthria, and left eyelid ptosis. He had dysphagia, axial and proximal motor weakness (Medical Research Council grade 4/5 in the upper limbs [8], right greater than left), without sensory deficit or pyramidal signs.

Timeline

**Figure 1:**
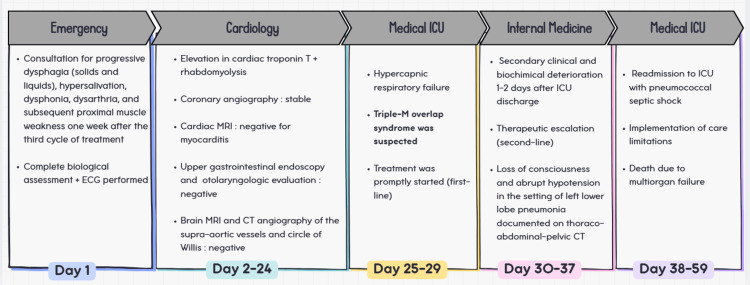
Timeline of the patient’s clinical course (days 1–59) Image credit: Created by the authors using Canva (Canva Pty Ltd., Sydney, Australia).

The timeline of the patient’s clinical course, from initial neuromuscular symptoms to fatal pneumococcal septic shock (Figure 1).

Diagnostic assessment

irAEs were suspected based on clinical and biochemical profile after atezolizumab (1200 mg) plus bevacizumab (15 mg/kg), including the following:

Immune-Related Myocarditis

**Figure 2:**
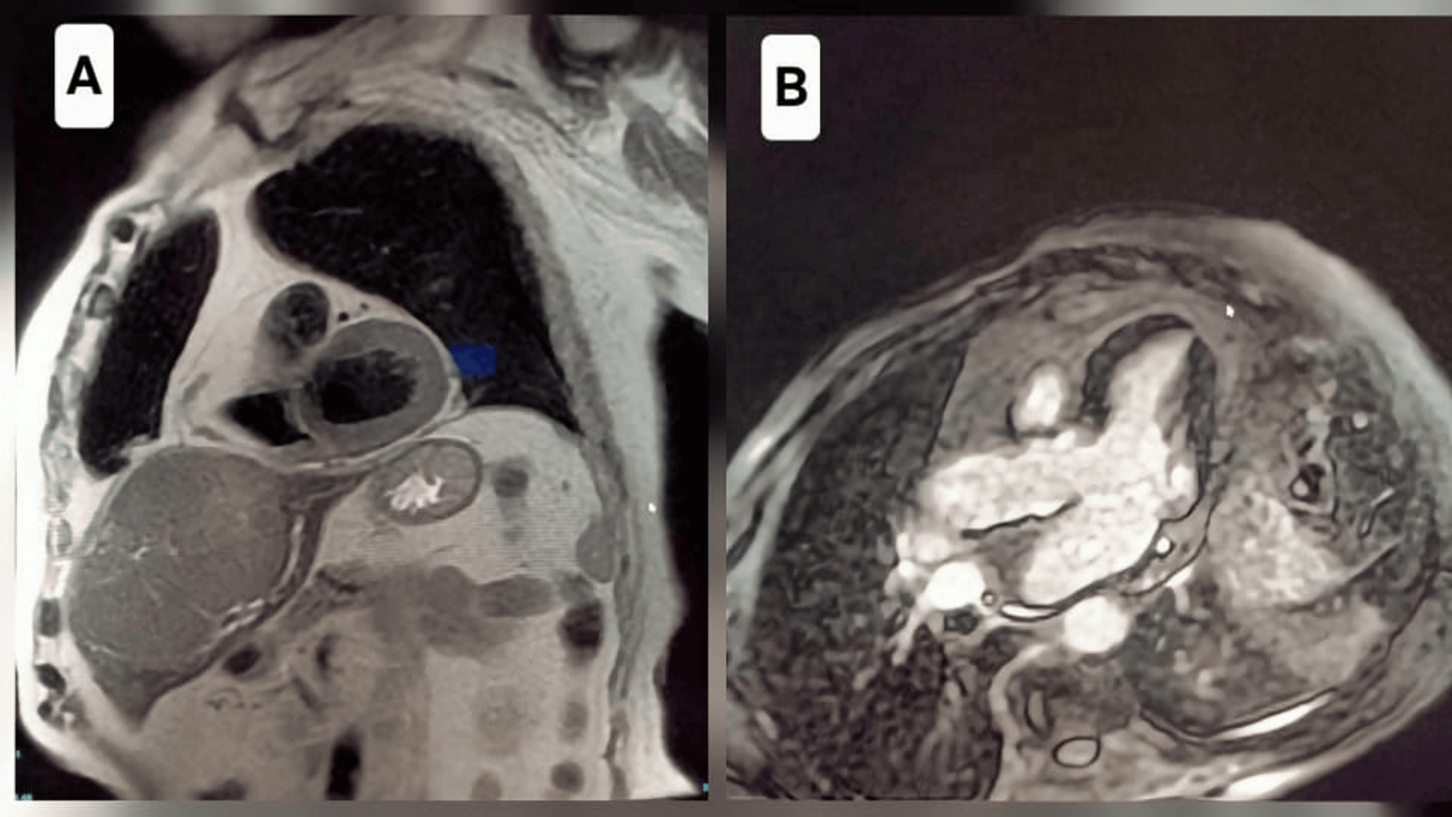
Cardiac MRI (A) T2 black-blood sequence, short axis view: homogeneous myocardial signal, without focal or diffuse hypersignal suggestive of myocardial edema. No inflammatory wall thickening or segmental signal abnormality; (B) Late gadolinium enhancement (LGE) sequence, three-chamber view: no focal or diffuse late myocardial enhancement, particularly in the subepicardial or intramural areas. There was no evidence of myocardial necrosis or inflammatory fibrosis.

There was persistent elevation of hs-cTnT, despite stable coronary angiography. The electrocardiogram (ECG) revealed a left bundle branch block pattern with secondary repolarization abnormalities, and transthoracic echocardiography showed left ventricular ejection fraction (LVEF) of 50% with known inferobasal/septal akinesia and no significant valvular disease. Cardiac MRI was negative for myocarditis according to the modified Lake Louise criteria (Figure 2).

Myositis

**Figure 3:**
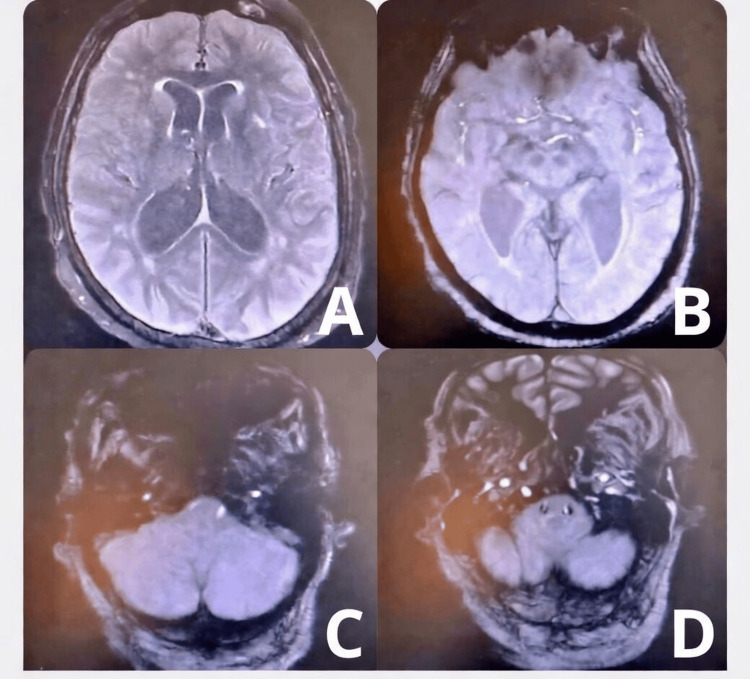
Brain MRI, axial slices (A) High-supratentorial T1 sequence, lateral ventricles symmetrical with preserved morphology; (B) Mid-supratentorial T2-weighted Fluid-Attenuated Inversion Recovery (T2/FLAIR) sequence, symmetrical deep structures and median third ventricle not dilated; (C) Low-supratentorial T2 sequence, apparent integrity of cerebral parenchyma; (D) Low-supratentorial T2 sequence, cerebellum with preserved morphology, symmetrical, visible fourth ventricle not compressed and brainstem with regular appearance. No abnormalities, particularly of the brainstem, explaining the symptoms.

Rhabdomyolysis pattern was observed with CK up to 1900 U/L (Upper limit of normal (ULN) <195 U/L, Aspartate Aminotransferase (AST)/Alanine Aminotransferase (ALT) 294/260 U/L (ULN <50 U/L for both), and lactate dehydrogenase (LDH) 655 U/L (reference range 125-250 U/L), associated with bulbar symptoms (dysarthria, dysphonia, dysphagia) without abnormalities on the brain MRI (Figure 3).

Myasthenia-like Involvement

There was presence of asymmetric proximal muscle weakness, left eyelid ptosis, respiratory muscle weakness, and bulbar symptoms. Acetylcholine receptor (AChR) antibodies were tested and were negative.

Diagnosis

Myocarditis-myositis-myasthenia-like overlap syndrome (Triple-M overlap syndrome).

Therapeutic interventions

Treatment was initiated promptly after presumptive diagnosis of severe irAEs.

Initial Phase

High-dose corticosteroids: intravenous methylprednisolone 1 g/day for three days, followed by gradual taper with oral prednisone at 1 mg/kg/day.

Intravenous immunoglobulin (IV Ig): total dose of 2 g/kg administered over five days by slow infusion.

Respiratory support: bilevel noninvasive ventilation (NIV) with individualized initial expiratory pressure settings, resulting in rapid correction of hypercapnia and complete weaning from NIV and supplemental oxygen before discharge from the first ICU phase.

Empiric antibiotic therapy: intravenous amoxicillin-clavulanate for CT-documented pneumonia, maintained during the high-dose steroid period.

Prophylaxis: trimethoprim-sulfamethoxazole for Pneumocystis jirovecii prophylaxis during prolonged corticosteroid therapy.

Secondary Clinical Deterioration

In the setting of rising hs-cTnT and CK levels with worsening motor deficit, intravenous corticosteroids were re-escalated (reintroduction of methylprednisolone at 1 mg/kg/day IV), and ruxolitinib was added at 5 mg two times per day for a total of six days, before discontinuation due to pneumococcal septic shock requiring intensive care.

Follow-up and outcomes

**Figure 4:**
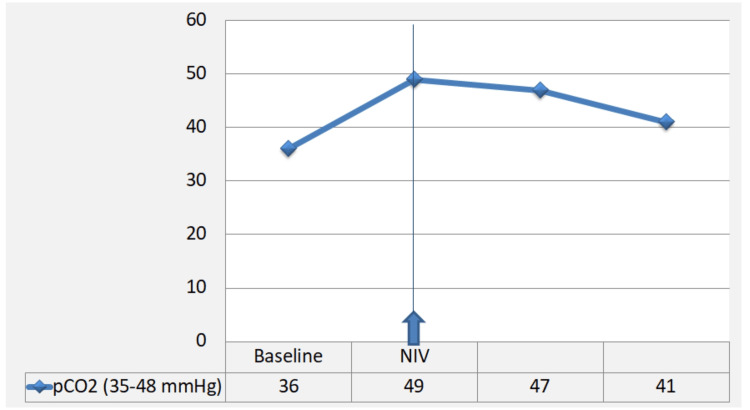
Evolution of partial pressure of carbon dioxide (pCO₂) during treatment An increase in pCO₂ was observed from baseline to noninvasive ventilation (NIV) initiation, which coincided with the start of first-line therapy. Thereafter, pCO₂ values progressively decreased under treatment, indicating an improvement in the ventilatory status.

**Figure 5:**
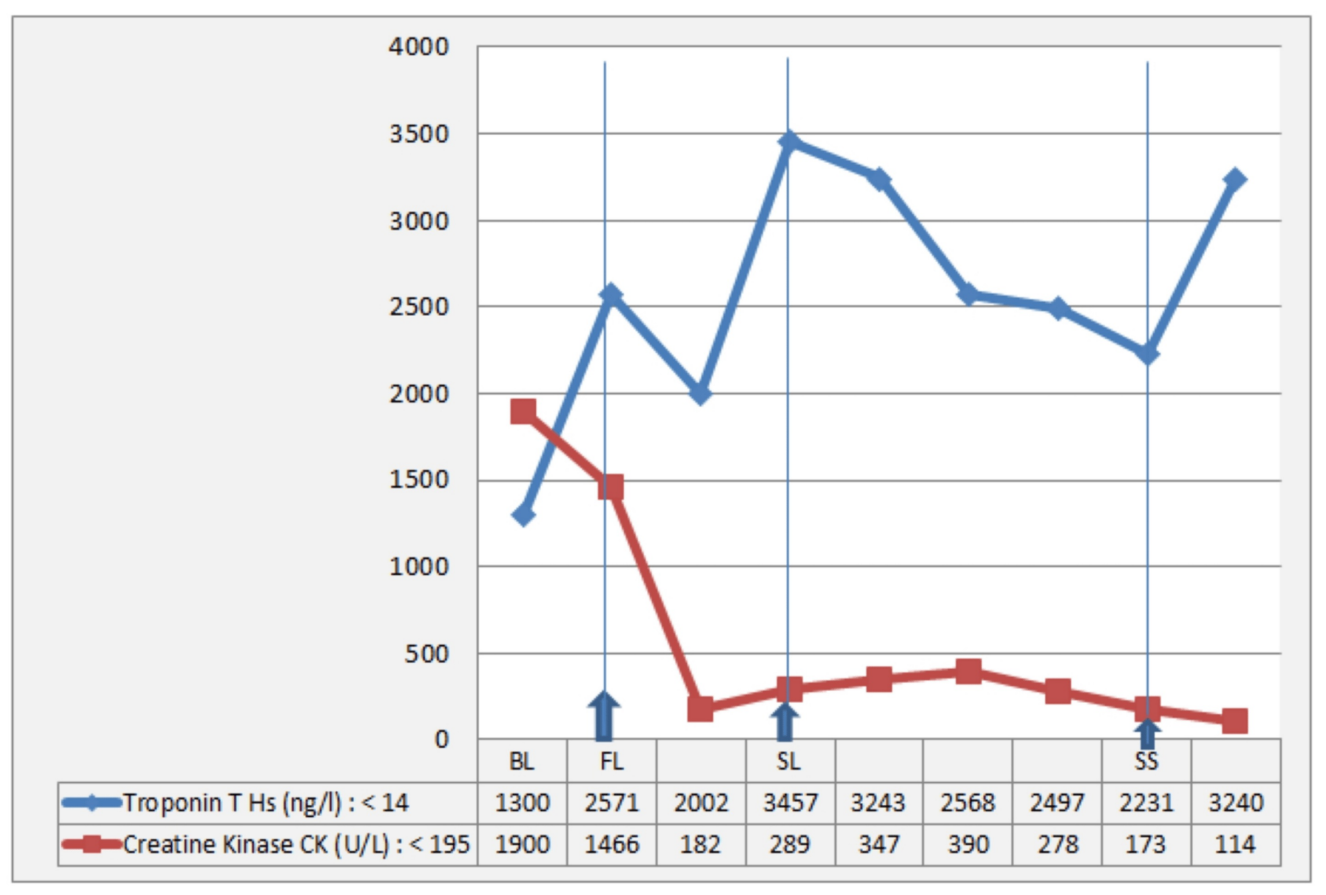
Temporal trend of cardiac and skeletal muscle biomarkers during management High-sensitivity cardiac troponin T (hs-TnT, ng/L; blue curve) and creatine kinase (CK, U/L; red curve) are shown at key clinical time points: baseline (BL), first-line treatment (FL), second-line treatment (SL), and septic shock (SS). Both biomarkers were markedly elevated at BL, followed by a significant decline after initiation of first-line and then second-line therapy. A subsequent rise in troponin was observed during the septic shock episode.

A marked initial response was observed under corticosteroids plus IV Ig with rapid normalization of blood gases, successful discontinuation of NIV, improvement in motor strength on serial neurological examination, and significant biomarker decline, with CK decreasing from 1466 to 182 U/L and troponin decreasing from 2571 to 2002 ng/L (Figures 4, 5).

Secondary clinical worsening occurred, with re-elevation of biomarkers and progression of motor weakness, requiring re-escalation to intravenous corticosteroids and addition of oral ruxolitinib. Subsequent complications included pneumococcal sepsis, leading to septic shock with multiorgan failure, culminating in death on September 2025.

Consent

Consent for publication of this anonymized case report was obtained in accordance with ethical standards.

## Discussion

This case highlights severe cardiac and skeletal muscle immune-related toxicity occurring during first-line atezolizumab-bevacizumab therapy for advanced HCC, with a clinicobiological profile consistent with probable myocarditis, inflammatory myositis with predominant bulbar involvement, and myasthenia-like syndrome. It illustrates a major real-world diagnostic challenge, particularly in a patient with pre-existing ischemic heart disease, and underscores the need for early recognition of this rare but potentially fatal presentation.

ICI-associated myocarditis is uncommon (estimated incidence, 0.3%-1.0%) but carries high mortality, reported at approximately 30%-40% in early cohorts [3,4]. Although more frequently described with PD-1 inhibitors and combination ICI regimens, accumulating evidence confirms that PD-L1 inhibitors, including atezolizumab, can also cause severe myocarditis, both as monotherapy and in combination with bevacizumab [9,10]. Current European cardio-oncology guidance states that any unexplained troponin elevation in a patient receiving ICI therapy should raise suspicion for myocarditis until proven otherwise, regardless of echocardiographic or cardiac MRI findings [11]. Cardiac MRI provides a noninvasive diagnostic framework for acute myocarditis using the modified Lake Louise criteria, typically requiring evidence of myocardial edema (T2-based abnormality) together with a non-ischemic injury pattern on T1/late gadolinium enhancement (LGE) imaging [12]. However, its sensitivity is limited (around 30% false negatives or partial), so a negative cardiac MRI result does not exclude the diagnosis when clinical suspicion is high [11]. Endomyocardial biopsy remains the diagnostic gold standard [3,4,6,10,11]. In practice, biopsy is an invasive procedure; it is rarely performed on an emergency basis and is reserved for ambiguous and persistent cases, especially in the event of negative imaging or to guide therapeutic escalation [6,10,11]. ICI myocarditis may also present with atypical features such as ventricular rhythm disorders and segmental hypokinesia with preserved LVEF [3,10]. In up to 50% of cases, no significant impairment of left ventricular (LV) systolic function is found, greatly limiting the sensitivity of echocardiography alone as a screening tool [6]. In our patient, persistent and marked elevation of hs-cTnT, together with unchanged coronary angiography excluding an acute coronary syndrome, strongly supported an immune-mediated myocarditis in this context, despite negative cardiac MRI and lack of histologic confirmation.

The major CK elevation observed in this case, accompanied by increased LDH and transaminases, was highly suggestive of immune-mediated inflammatory myositis. ICI-related myositis is now well characterized and may present with proximal limb weakness as well as bulbar striated muscle involvement, causing dysphagia, dysarthria, or dysphonia [6,9,10]. Several reports under atezolizumab, including in combination with bevacizumab, have described similar presentations with prominent dysphagia and CK elevation [6,12,13]. In most typical cases, diagnosis is made clinically and biochemically without routine muscle biopsy, and treatment is started promptly, unlike myocarditis, where endomyocardial biopsy is more frequently debated, though still not systematically performed [5,6,9-13]. These symptoms are crucial to recognize because they may precede or obscure concomitant cardiac toxicity. In our patient, inaugural dysphagia with dysphonia and dysarthria, in the absence of identifiable otorhinolaryngology or digestive structural pathology, together with proximal (albeit asymmetric) muscle weakness, strongly supported major neuromuscular involvement. Electromyography was not performed in our case. The coexistence of myocarditis and myositis in this patient is consistent with the increasingly recognized Triple-M overlap syndrome reported with ICIs [5,6,9,10,13,14]. In our case, myasthenia could not be confirmed serologically because AChR antibodies were negative; however, the clinical phenotype of left ptosis, asymmetric proximal weakness, and respiratory failure suggestive of muscle fatigue was strongly supportive of myasthenia-like involvement. Importantly, AChR antibody positivity appears lower in ICI-related myasthenic syndromes than in idiopathic myasthenia gravis (approximately 60% vs 85%), so seronegativity does not exclude the diagnosis [6,10].

From a therapeutic perspective, immediate ICI discontinuation and early initiation of high-dose systemic corticosteroids (methylprednisolone 500-1000 mg/day) are recommended once clinical suspicion is high, without waiting for histologic confirmation or advanced imaging, given the substantial mortality risk [5,6,9,11,13-15]. In cases with bulbar neuromuscular involvement, as in our patient, close respiratory monitoring is essential, and additional immunomodulatory therapies such as IV Ig or plasmapheresis should be considered on an individualized basis [5,6,13,14]. For steroid-refractory immune toxicities, combination strategies including corticosteroids plus ruxolitinib may be considered. Ruxolitinib, a selective Janus kinase (JAK)1/2 inhibitor targeting the JAK-STAT inflammatory pathway, is emerging as a potential rescue option with reported clinical and biochemical benefit, despite the increased risk of infection associated with its use [16,17]. Published data indicate that overlap syndrome is associated with higher severity and poorer prognosis, with increased risks of respiratory failure, malignant arrhythmias, and death [5,6,13,14]. In our patient, first-line therapy (high-dose intravenous corticosteroids followed by oral taper, intravenous immunoglobulin, and NIV for hypercapnic respiratory failure) led to initial improvement (Figures 4, 5). Subsequent re-elevation of troponin and CK, together with worsening motor deficit, prompted second-line escalation with reintroduction of intravenous corticosteroids and addition of ruxolitinib. Management decisions were made within a multidisciplinary framework involving oncology, cardiology, intensive care, internal medicine, and neurology teams. Although biomarker kinetics improved thereafter, the patient ultimately died from pneumococcal septic shock of pulmonary origin with multiorgan failure.

Despite the lack of histological confirmation of myocarditis and electromyogram for musculoskeletal involvement, this case illustrates the importance of multidisciplinary management and therapeutic intensification in cases of severe immune toxicities under ICIs. The combination of high-dose corticosteroids, IV Ig, and ruxolitinib resulted in significant biological and clinical improvement, suggesting that rapid intervention can greatly improve prognosis and potentially be life-saving, while taking into account the risk of infection. Our patient died of septic shock, probably related to the immunosuppression induced by the treatment of this syndrome.

## Conclusions

This case highlights the Triple-M overlap syndrome induced by the combination of atezolizumab and bevacizumab, a rare but serious immunological adverse effect involving cardiac, neuromuscular, and respiratory damage, which is difficult to diagnose and must be considered early on, despite negative cardiac MRI results sometimes.

Management is based on rapid discontinuation of immunotherapy and a multidisciplinary approach. In severe or refractory forms, ruxolitinib may be considered as a rescue treatment, with a careful assessment of the risk of infection.
